# Niche, not phylogeny, governs the response to oxygen availability among diverse *Pseudomonas aeruginosa* strains

**DOI:** 10.3389/fmicb.2022.953964

**Published:** 2022-08-17

**Authors:** Sonal Shewaramani, Rees Kassen

**Affiliations:** Department of Biology, University of Ottawa, Ottawa, ON, Canada

**Keywords:** *Pseudomonas aeruginosa*, oxidative stress, phylogenetic relatedness, trait responses, ecological niches

## Abstract

*Pseudomonas aeruginosa*, a ubiquitous opportunistic pathogen, is a leading cause of chronic infection of airways in cystic fibrosis (*CF*) patients. Chronic infections typically arise from colonization by environmental strains, followed by adaptation of *P. aeruginosa* to the conditions within the *CF* airway. It has been suggested that oxygen availability can be an important source of selection causing trait changes associated with the transition to chronic infection, but little data exist on the response of *P. aeruginosa* to varying levels of oxygen. Here, we use a diverse collection of *P. aeruginosa* strains recovered from both *CF* patients and environmental sources to evaluate the role of oxygen availability in driving adaptation to the *CF* lung while also accounting for phylogenetic relatedness. While we can detect a signal of phylogeny in trait responses to oxygen availability, niche of origin is a far stronger predictor. Specifically, strains isolated from the lungs of *CF* patients are more sensitive to external oxidative stress but more resistant to antibiotics under anoxic conditions. Additionally, many, though not all, patho-adaptive traits we assayed are insensitive to oxygen availability. Our results suggest that inferences about trait expression, especially those associated with the transition to chronic infection, depend on both the available oxygen and niche of origin of the strains being studied.

## Introduction

The bacterium *Pseudomonas aeruginosa* is a ubiquitous, Gram-negative, opportunistic pathogen often found in association with a wide range of ecological niches, including environmental sources such as soil and water or human and plant hosts (Rajan and Saiman, 2002; Folkesson et al., 2012). In humans, *P. aeruginosa* is a common cause of acute and chronic infections in patients with cystic fibrosis (*CF*). Once established in *CF* airways, chronic *P. aeruginosa* infections are persistent and difficult to eradicate, making them the leading cause of morbidity and mortality in adult patients (Aaron et al., 2010). Although highly transmissible epidemic strains exist, the majority of chronic infections arise *via* colonization of airways with *P. aeruginosa* acquired from environmental sources (Folkesson et al., 2012; Rossi et al., 2021). The transition from environmental strain to chronic infection in *CF* patients is accompanied by multiple phenotypic changes evolving through repeated, convergent evolution by diverse colonizing strains as they adapt to the uniquely stressful conditions of the *CF* lung. In contrast to environmental strains, isolates of *P. aeruginosa* obtained from the airways of chronically infected *CF* patients are typically non-motile, mucoid, highly antibiotic resistant and avirulent (Smith et al., 2006; Folkesson et al., 2012; Sousa and Pereira, 2014; Sousa et al., 2018; Rossi et al., 2021). These trait changes are thought to be underpinned by a suite of genetic changes resulting from within-host, mutation-driven evolution over the course of an infection (Smith et al., 2006; Folkesson et al., 2012).

The environmental sources of selection responsible for the transition from environmental strain to chronic endobronchial infection in *CF* patients remain poorly understood. Colonizing *P. aeruginosa* populations encounter complex and challenging conditions in *CF* airways that they must overcome to persist and survive. The *CF* lung is characterized by osmotic, oxidative and nitrosative stress, limited nutrient and oxygen availability, high concentrations of antibiotics, constant immune system attack and the presence of other competing microorganisms (Filiatrault et al., 2006; Folkesson et al., 2012; Sousa and Pereira, 2014; Botelho et al., 2019). As such, cellular pathways implicated in the adaptation of *P. aeruginosa* to *CF* airways often include those involved in stress responses, respiration and energy production, antibiotic metabolism, pathogenesis and transport (Dettman et al., 2013), a result confirmed by a recent comparative genomic analysis of 1,000 *P. aeruginosa* strains that identified genetic targets of niche-specific adaptation associated with *CF* isolates (Dettman and Kassen, 2021). Of particular note was that many genes associated with regulating oxidative stress and the maintenance of redox homeostasis in response to oxidative stress were found to be under stronger positive selection in the *CF* airway compared to non-*CF* environments. Taken together, these results suggest that oxidative stress is a key factor contributing to adaptive evolution of *P. aeruginosa* within *CF* airways.

Evaluating this hypothesis has been challenging for two reasons. First, there is no widely accepted animal or laboratory model that recapitulates the complex conditions of *CF* airways. The development of a synthetic *CF* medium (SCFM), a defined medium designed to mimic the nutritional complexity of the *CF* lung (Palmer et al., 2007), has been a major victory on this front, but much work remains. *In vitro* studies with this medium, for example, do not capture other important dimensions of the *CF* airway such as competing microflora and the host immune response (Folkesson et al., 2012; Rossi et al., 2021). Second, one environmental characteristic thought to be important in the *CF* lung, oxygen levels, has received little attention from laboratory studies. Conventional phenotypic characterization of *P. aeruginosa* strains involves analyzing planktonic cultures of *P. aeruginosa* under fully oxic conditions, where they can utilize oxygen for aerobic respiration. However, *CF* airways contain steep oxygen gradients resulting from thick mucus layers, biofilms and/or the consumption of oxygen by epithelial and immune cells (Worlitzsch et al., 2002). The response of *P. aeruginosa* to these conditions has not been investigated, though there are a few studies investigating the effect of growth under microaerophilic or anaerobic conditions on the antimicrobial susceptibility of clinical strains of *P. aeruginosa* (Yoon et al., 2002; Field et al., 2005; Hill et al., 2005; Chandler et al., 2019). Consequently, we are still some way from a complete understanding of the spectrum of selective conditions and phenotypic responses of *P. aeruginosa* in the *CF* lung.

As a facultative anaerobe, *P. aeruginosa* is metabolically versatile and can grow in the presence or absence (anoxia) of oxygen. Under anoxic conditions, *P. aeruginosa* can achieve rapid growth through the use of nitrate or nitrite as an alternative terminal electron acceptor during anaerobic respiration (Hassett et al., 2002). Alternatively, *P. aeruginosa* growth may be slow, as it generates energy *via* substrate-level phosphorylation of arginine or pyruvate during fermentation (Filiatrault et al., 2006; Schreiber et al., 2006). Both *CF* mucus and sputum have been found to contain nitrate, nitrite and arginine in sufficient quantities, indicating that *P. aeruginosa* growth without oxygen in the *CF* lung is possible (Hassett et al., 2002; Worlitzsch et al., 2002). Characterizing the phenotypes of *P. aeruginosa* clinical strains under oxygen conditions more similar to *CF* airways is a necessary step in gaining a thorough understanding of the traits expressed by these strains in the lung itself.

To gain a better understanding of the role of oxidative stress in driving the adaptive evolution of *P. aeruginosa* within *CF* lungs, we measured the tolerance of *P. aeruginosa* strains isolated from distinct ecological niches to different oxidative stressors under a range of oxygen conditions. Assays were conducted in conditions that mimic the nutrient conditions of the *CF* lung by using SCFM across three oxygen levels: an environment with atmospheric oxygen, an environment with low levels of oxygen (microaerophilic) and an environment with no oxygen (anoxic). We focus on putatively patho-adaptive traits including resistance to two classes of antibiotics, swim and twitch motility, pyocyanin and pyoverdine production and biofilm formation, as their response to varying levels of oxygen in the context of chronic *CF* lung infections has not, to our knowledge, been studied.

We designed our experimental work with two objectives in mind. The first is to test the hypothesis that oxidative stress can be a major source of selection in the *CF* lung and the second is to evaluate the impact that oxygen availability has on trait expression in *P. aeruginosa*. Strong inferences require evaluating trait responses for strains from diverse origins that include both the *CF* airway and the environment. The rationale behind this approach is that *P. aeruginosa* is such an ecologically and genetically diverse group that the usual practice in microbiology of taking one or two strains (most commonly PA01 and PA14) to be representative of the group as a whole could skew our results. Our approach requires we account explicitly for phylogeny in our analyses in order to distinguish whether the phenotypic responses we observe are due to shared inheritance from a common ancestor or repeated evolution of the same traits from different ancestors (also known as convergent evolution). An observation of repeated evolution would lend support to the idea that selection associated with oxidative stress is an important driver of adaptation to the *CF* lung.

## Materials and methods

### Bacterial strains and growth media

We screened a diverse collection of *P. aeruginosa* strains (Supplementary Table 1) that includes eight strains isolated from different chronically infected *CF* airways (clinical sources) and nine strains isolated from the environment (Dettman and Kassen, 2021). The two most commonly used laboratory strains, PAO1 and PA14, were also included to allow comparison with other studies (Supplementary Table 2). For each strain, cultures from frozen stocks were plated on Lysogeny Broth (LB) agar plates and incubated at 37°C for 24 h under oxic conditions, after which two isolates were randomly picked per strain. All isolates were subsequently grown overnight in LB at 37°C and shaken at 150 RPM, after which they were stored at −80°C in 20% glycerol.

### Phenotypic assay growth conditions

Isolates (two per strain) were screened in all three oxygen environments using standard phenotype assays, described in detail below. Assays were performed in quadruplicate unless specified otherwise for a total of 152 samples per environment for each phenotype. All cultures were propagated in SCFM (Palmer et al., 2007) supplemented with 10 mM KNO_3_ and incubated for 24 h at 37°C and shaken at 150 RPM unless otherwise indicated. While SCFM medium is designed to mimic the nutritional complexity of the *CF* lung, supplemental nitrate was added to media to support growth of strains under anaerobic conditions (Palmer et al., 2007), such that cell densities of at least 10^7^ cells/mL were reached for all strains within 24 h of growth. All anaerobic media was prepared by placing aerobically prepared media in an anaerobic glove box for 24 h before use.

Isolates were grown overnight by inoculating frozen stocks into wells of 24-well plates containing 1.5 ml of SCFM media. Strains were acclimated to their growth environment for 24 h by transferring 1% of an overnight culture into fresh SCFM medium. Microaerophilic populations were incubated in AnaeroPack™ 7 l gas boxes along with AnaeroPouch™-MicroAero Gas Generator sachets to maintain a low oxygen environment. Anoxic populations were propagated in an anaerobic glove box containing a 95% N_2_:5% H_2_ atmosphere and placed in AnaeroPack™ 7 l gas boxes to maintain an anaerobic atmosphere after removal from the chamber, along with oxygen indicator strips to monitor oxygen conditions.

### Oxidative stress resistance

Resistance to two stressors, hydrogen peroxide and sodium nitrite, was measured by determining the lowest concentration that prevents 90% of growth as determined by optical density at 600 nm. These minimum inhibitory concentrations (MICs) were measured in 200 μl of medium by using a twofold dilution series of each chemical and 1:1000 diluted growth environment acclimated culture. Concentrations ranged from 0 mM to 250 mM and 0 mM to 240 mM for hydrogen peroxide and sodium nitrite, respectively.

### Antimicrobial resistance

Resistance to two commonly used antibiotics, ciprofloxacin and tobramycin, was measured by determining their MIC as described above. Concentrations ranged from 0 μg/ml to 8 μg/ml and 0 μg/ml to 16 μg/ml for ciprofloxacin and tobramycin, respectively.

### Motility assays

Twitch and swim motility assays were measured as described in (Clark et al., 2015). First, frozen stocks of isolates were plated on 1.5% LB agar plates to isolate individual colonies. We then randomly chose four colonies from each isolate that were stab-inoculated into 1.5% LB agar plates supplemented with 10 mM KNO_3_ and incubated for 48 h at 37°C. Agar was subsequently removed from the petri dish and plates were stained with a 0.1% crystal violet in water solution for 5 min. Excess dye was then rinsed off with water and the zone of growth (in mm) was measured for each colony. To measure swim motility, colonies were stab-inoculated into 0.3% LB agar plates supplemented with 10 mM KNO_3_. Plates were incubated for 24 h at 37°C and the zone of growth (in mm) was measured for each colony.

### Biofilm formation

Biofilm formation was assayed as described by (O’Toole, 2011). Growth environment acclimated cultures were diluted 1:100 in fresh growth medium and 100uL of each culture was seeded into wells of 96-well non-tissue culture treated microtiter plate (Corning). Cultures were incubated at 37°C for 24 h under static conditions, after which their optical density was measured at 600 nm to estimate cell density. Plates were then rinsed with water and stained with 125 μl of a 0.1% crystal violet in water solution. Plates were incubated at room temperature for 20 min, rinsed with water several times to remove excess dye, and then dried for 48 h. When plates were dry, 125uL of 30% acetic acid in water was used to dissolve the dye and plates were incubated at room temperature for 20 min. Cultures were then transferred to fresh plates and the optical density of the solution at 550 nm was read. Biofilm formation was quantified by standardizing the solubilized biofilm measurement by the respective initial cell density of that well (i.e., OD_550_/OD_600_).

### Virulence factor production

To measure virulence factor production, the optical density of growth environment acclimated cultures was measured at 600 nm. Additionally, 1 ml from each culture was centrifuged at 8000 RPM for 10 min. Supernatant from each culture was read at 405 nm and 695 nm for pyoverdine and pyocyanin, respectively. Pyoverdine and pyocyanin formation was quantified by standardizing the supernatant measurements by the respective cell density.

### Phylogeny

To explore the evolutionary relationship between isolates and the phenotypes discussed above, we inferred the phylogenetic relationship among strains using whole-genome sequences to identify a core genome shared among all isolates. Using PA01 as the reference genome and PA7 (RefSeq accession: GCF_000017205.1) as an outgroup, Prokka (Seemann, 2014) was used to annotate genomes and Get Homologues (Contreras-Moreira and Vinuesa, 2013) with default parameters was used to identify the core genome. The core genome consisted of 3,606 genes shared by all isolates. Core gene sequences were then aligned with MAFFT 7.471 (Katoh and Standley, 2013) with default parameters and a maximum likelihood phylogenetic tree was inferred with the software selected GTR + F + I + G4 substitution model and 1,000 ultrafast bootstrap (UFBoot) replicates for branch support using IQ-TREE-2.0.7 (Minh et al., 2020). The phylogenetic tree was visualized with either the interactive tree of life (iTOL) webtool (Letunic and Bork, 2021) or using the ape package in R (R Core Team, 2018).

### Statistical analyses

All statistical analyses were conducted using R version 4.0.0 (R Core Team, 2018). All phenotypic data was treated as continuous variables and log-transformed in order to obtain residuals with distributions close to Gaussian, which were confirmed for all response variables after visual inspection of model residuals. Figures were generated using the R ggplot2 package (Wickham, 2016).

To determine the effect of niche, growth environment, and their interaction on each phenotype individually, we used the R package MCMCglmm to construct phylogenetic Markov Chain Monte Carlo generalized linear mixed effects models (Hadfield, 2010). Each phenotype was fitted as a Gaussian response variable with strain niche (clinical or environmental), growth environment (oxic, microaerophilic or anoxic) and the niche by environment interaction as fixed effects. Individual genotypes and their interaction with the growth environment were modeled as random effects. Each model was run for 8,000,000 iterations with a burn-in of 700,000 and thinning interval of 1,000 to minimize any autocorrelation between posterior samples, giving an effective sample size of at least 1,000. Duplicate chains were run for each model and model convergence was checked by visually observing trace plots of MCMC chains, evaluating correlation between samples (autocorrelation <0.1) and using Gelman–Rubin tests (potential scale reduction factor (PSRF) < 1.1 among chains) from the R package coda (Plummer et al., 2006). Default normal priors were used for fixed effects and for all random effects, inverse-gamma priors (*V* = 1, nu = 0.02) were used, which led to well-mixed chains with low autocorrelation. The parameter estimates from models are reported here as posterior means and 95% credible intervals (CIs); lower CI–upper CI. We then performed *post hoc* pairwise comparison tests to evaluate the statistical significance among fixed effects using the R package emmeans (Lenth, 2022). Parameter estimates were considered to be statistically significant when 95% CIs of the contrasts did not include zero. For each phenotype, two models were run; one accounted for phylogenetic relatedness between strains, while the other did not (Supplementary Table 3). To account for the relatedness among strains, we used our phylogeny to create a phylogenetic covariance matrix which was then included as a random effect in the model. Phylogenetic heritability (*H*^2^) was calculated as the proportion of residual variation explained by phylogenetic covariance and is comparable to Pagel’s *λ*, as the phylogeny is integrated within the model itself.

## Results

### Clinical and environmental strains are distributed evenly across the phylogeny

**Figure 1:**
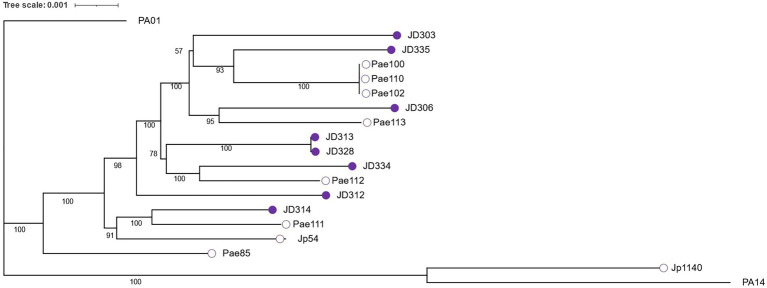
Maximum likelihood phylogeny constructed from the alignment of 3,606 core genes and visualized with iTOL (Letunic and Bork, 2021). Bootstrap values (50%–100%) for internal branches are indicated in black. Empty circles depict environmental strains while shaded purple circles depict clinical strains.

Based on a 3,606 core genome alignment, we constructed a maximum likelihood phylogeny of the *P. aeruginosa* strains included in this study (Figure 1). Consistent with previously published phylogenies (Dettman et al., 2013; Freschi et al., 2018; Dettman and Kassen, 2021), the commonly used lab strains PA01 and PA14 fall into different clades in our phylogeny. In the subset of strains chosen for this study, little association was found between phylogenetic structure and ecological source. Clinical and environmental strains were dispersed fairly evenly across the phylogeny, consistent with findings from a larger dataset (Dettman and Kassen, 2021). This result indicates that isolates from the same ecological niche can belong to different clades within the phylogeny, supporting the inference from previous work (Dettman and Kassen, 2021) that any strain or clade can develop into a chronic *CF* infection.

### Clinical strains are more sensitive to external oxidative stress than environmental strains

**Figure 2:**
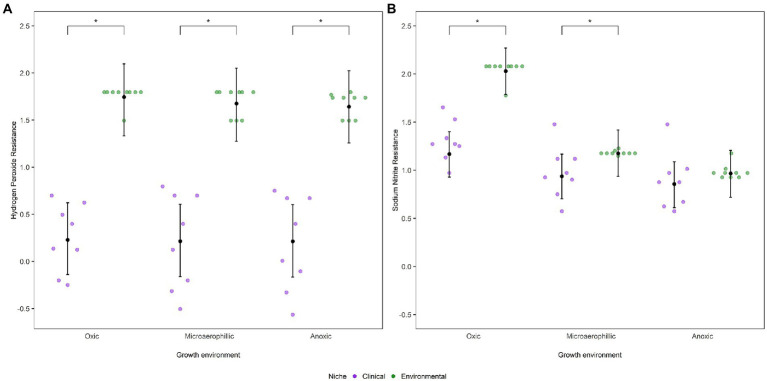
Dot plots represent mean MIC values (log_10_ transformed) of *P. aeruginosa* strains as a function of niche and environment for oxidative stressors. **(A)** Hydrogen peroxide and **(B)** Sodium nitrite. The posterior mean and 95% CIs are indicated by the black dot and whiskers, respectively, as calculated from the phylogenetic mixed model as determined by MCMCglmm. Statistically significant differences, *α* < 0.05, between niches as estimated by emmeans are depicted by *.

Strains isolated from both clinical and environmental sources were assessed for their resistance to oxidative stress by measuring their sensitivity to two chemicals that induce oxidative stress: hydrogen peroxide (H_2_O_2_) and sodium nitrite (NaNO_2_). Previous genomic analyses showed that genes associated with redox functions are under relatively stronger positive selection in *CF* isolates than in environmental isolates (Dettman et al., 2013; Dettman and Kassen, 2021). We therefore expected that isolates from clinical sources would be able to withstand greater concentrations of external oxidative stressors than isolates from environmental sources, especially when grown under microaerophilic or anoxic conditions. Our results, however, do not match this expectation: isolates from environmental sources were 4-fold more resistant, on average, to hydrogen peroxide than clinical strains (Figure 2A). This surprising result was robust to the inclusion of phylogeny into the statistical model (Supplementary Table 3), as the phylogenetic signal for this trait was negligible (*H*^2^ = 0.08, 95% CI: 0.03–0.84). Resistance to hydrogen peroxide across ecological niches was not influenced by oxygen availability, as the difference between environmental and clinical strains was consistent across all three oxygen environments (*Β* = 1.43, 95% CI: 1.10–1.77). Resistance to hydrogen peroxide within clinical strains was also not influenced by oxygen availability, though environmental strains were generally less resistant to hydrogen peroxide under anoxic conditions than oxic conditions (Supplementary Table 4).

For sodium nitrite, environmental strains were, on average, 1.5-fold more resistant than clinical strains under oxic conditions (Figure 2B). Incorporating phylogeny into the analysis yielded comparable results (Supplementary Table 3), despite a moderate phylogenetic signal (*H*^2^ = 0.25, 95% CI: 0.04–0.68). However, resistance to sodium nitrite was influenced by oxygen availability: under microaerophilic conditions, environmental strains were slightly more resistant than clinical strains (*Β* = 0.24, 95% CI: 0.03–0.45) while under anoxic conditions we did not detect a difference between the resistance of clinical and environmental strains (*Β* = 0.11, 95% CI: −0.10–0.33). Even within each niche, resistance to sodium nitrite was influenced by oxygen availability; clinical strains were more susceptible to sodium nitrite under anoxic and microaerophilic conditions than under oxic conditions while environmental strains were less resistant to the stressor as oxygen levels decreased (Supplementary Table 4).

### Clinical strains are highly resistant to antibiotics under anoxic conditions

**Figure 3:**
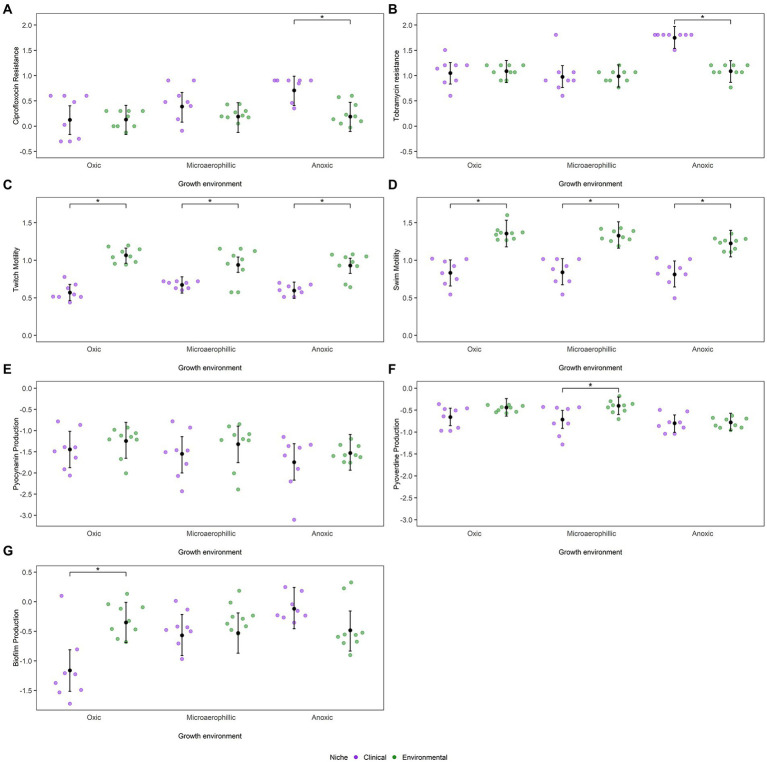
Dot plots represent mean values (log_10_ transformed) of *P. aeruginosa* strains as a function of niche and environment for **(A)** ciprofloxacin MIC, **(B)** tobramycin MIC, **(C)** twitch motility, **(D)** swim motility, **(E)** pyocyanin production, **(F)** pyoverdine production and **(G)** biofilm formation. The posterior mean and 95% CIs are indicated by the black dot and whiskers, respectively, as calculated from the phylogenetic mixed model as determined by MCMCglmm. Statistically significant differences, *α* < 0.05, between niches as estimated by emmeans are depicted by *.

Strains isolated from both clinical and environmental sources were also assessed for their resistance to two commonly used antibiotics with different modes of action, the quinolone ciprofloxacin and the aminoglycoside tobramycin. We hypothesized that isolates from clinical sources would be able to withstand greater concentrations of both antibiotics than isolates from environmental sources. However, there were no significant differences in mean ciprofloxacin resistance among clinical and environmental isolates under oxic and microaerophilic conditions (Figure 3A). Conversely, under anoxic conditions, clinical strains were 1.5-fold more resistant to ciprofloxacin than environmental strains (*Β* = −0.52, 95% CI: −0.79 to −0.22). While resistance to ciprofloxacin within environmental strains was not influenced by oxygen availability, clinical strains were generally more resistant to ciprofloxacin as available oxygen levels decreased (Supplementary Table 4). Additionally, a moderate phylogenetic signal was observed for ciprofloxacin resistance (*H*^2^ = 0.32, 95% CI: 0.04–0.61; Supplementary Table 3).

A similar trend was observed with the antibiotic tobramycin. There were no significant differences in mean tobramycin resistance among isolates from clinical and environmental sources under oxic and microaerophilic conditions (Figure 3B). Under anoxic conditions, however, clinical strains were 1.5-fold more resistant to tobramycin than environmental strains (*Β* = 0.66, 95% CI: 0.44–0.90). While resistance to tobramycin within environmental strains was not impacted by oxygen availability, clinical strains were more resistant to tobramycin as oxygen levels decreased (Supplementary Table 4). Incorporating phylogeny into the analysis yielded comparable results (Supplementary Table 3), despite a moderate phylogenetic signal (*H*^2^ = 0.18, 95% CI: 0.05–0.62).

### Differences in motility between clinical and environmental strains are insensitive to oxygen availability

As expected, clinical and environmental isolates significantly differed in their ability to move *via* twitch motility under oxic conditions, with environmental strains displaying approximately 3-fold more twitch motility than clinical strains (Figure 3C). These niche differences were consistent across all oxygen conditions (*Β* = 0.34, 95% CI: 0.19–0.49). Twitch motility within a niche, however, was affected by oxygen availability as environmental strains were less motile under anoxic and microaerophilic conditions than under oxic conditions (Supplementary Table 4). Phylogenetic position explained a significant proportion of variation in twitch motility (*H*^2^ = 0.34, 95% CI: 0.11–0.66, Supplementary Table 3). We found a similar trend for measurements of swim motility (Figure 3D). Clinical and environmental isolates significantly differed in their ability to move *via* swim motility under all oxygen conditions (*Β* = 0.41, 95% CI: 0.25–0.57). Once again, environmental strains were less motile under anoxic conditions than under oxic or microaerophilic conditions (Supplementary Table 4). As was observed with twitch motility, a strain’s phylogenetic position had some influence on its swim motility (*H*^2^ = 0.23, 95% CI: 0.06–0.60; Supplementary Table 3).

The siderophore pyoverdine is important for scavenging iron in low-iron environments, such as *CF* airways, while pyocyanin is a virulence factor associated with disease severity and lung function decline in *CF* patients (Tyrrell and Callaghan, 2016). After controlling for any phylogenetic dependency (Supplementary Table 3), differences in pyocyanin production across the two ecological niches under different oxygen conditions were trivial (Figure 3E). Pyocyanin production was impacted by oxygen availability with both environmental and clinical strains producing less pyocyanin as available oxygen levels decreased, though once again these differences were not significant within either niche (Supplementary Table 4). While pyocyanin virulence factor production had a negligible phylogenetic signal (*H*^2^ = 0.03, 95% CI: 0.01–0.61), pyoverdine production had a moderate phylogenetic signal (*H*^2^ = 0.18, 95% CI: 0.03–0.50). Clinical strains produced less siderophore (Figure 3F) than environmental strains under oxic and anoxic conditions (*Β* = 0.02, 95% CI: −0.20–0.25) though these difference in production were not significant. However, clinical strains produced significantly less pyoverdine than environmental strains under microaerophilic conditions (*Β* = −0.31, 95% CI: −0.54 to −0.10). Furthermore, environmental and clinical strains produced less pyoverdine under anoxic conditions than under oxic or microaerophilic conditions, though the differences in pyoverdine production were only significant for environmental strains (Supplementary Table 4). As the production of both of these pigments is highly dependent on oxygen availability to begin with, the decreased production of both pigments under anoxic conditions are not surprising (Visaggio et al., 2015; Castañeda-Tamez et al., 2018). Biofilm production, on the other hand, exhibited significant niche differences between clinical and environmental strains only under oxic conditions (Figure 3G), where environmental strains were better biofilm producers than clinical strains after accounting for differences in their initial cell densities (*Β* = 0.37, 95% CI: −0.02–0.75). However, while biofilm production within environmental strains was not impacted by oxygen availability, clinical strains produced more biofilm as available oxygen levels decreased (Supplementary Table 4). Accounting for phylogenetic relatedness yielded similar results as biofilm production had negligible phylogenetic signal (*H*^2^ = 0.04, 95% CI: 0.01–0.47; Supplementary Table 3).

## Discussion

Our study provides insight into the role that oxygen availability plays in governing the development of chronic infections of the *CF* airway by *P. aeruginosa.* We measured the response of a range of putatively patho-adaptive traits, including tolerance to known oxidative stressors, to varying levels of oxygen in a diverse collection of strains isolated from *CF* infections and the environment. Our leading results are that, after accounting for phylogenetic relatedness among our strains, clinical isolates were, on the whole, less tolerant of oxidative stress than environmental strains (Figures 2A,B) and that a number of traits associated with chronic infection show evidence of differential responses to oxygen availability. Together these results suggest that oxygen availability may be an important driver of selection in the *CF* lung, though not in the way anticipated by the results of comparative genomic surveys (Dettman et al., 2013; Dettman and Kassen, 2021).

### Why do *CF* isolates show reduced tolerance to oxidative stress despite evidence of strong positive selection in redox-associated genes?

Comparative genomic studies have revealed that genes associated with alleviating redox stress experience relatively stronger positive selection in *CF* isolates compared to acute or environmental isolates (Dettman et al., 2013; Dettman and Kassen, 2021), suggesting that *CF* strains have recently adapted to dealing with redox-associated stress. Strong selection for alleviating oxidative stress in the *CF* lung could come from three sources: reactive oxygen species stemming from respiration, host macrophage-derived oxidative burst, and antibiotics with redox-related mechanisms of action (Folkesson et al., 2012). Our experiments reveal, however, that *CF* isolates are actually *less* tolerant than environmental isolates to hydrogen peroxide and sodium nitrite-derived oxidative stress, in contrast to the inferences made from comparative genomic studies. What explains these contrasting results?

One possibility may be that the evidence for positive selection in redox-associated genes is driven not by redox stress itself but by some other stress we have not measured here. Many redox-associated genes also play a role in more general regulatory pathways responsible for responding to a range of stressors such as antibiotic resistance. For instance, *P. aeruginosa* in *CF* airways are also exposed to osmotic stress due to thick mucus layers within the lungs and electrolyte imbalances due to defective ion transport in the host (Aspedon et al., 2006; Folkesson et al., 2012; Rossi et al., 2021) and this could be why a signal was observed for some genes like *algU, osmC*, *oxyR* and *nirQ* in the comparative genomic analyses (Dettman and Kassen, 2021).

It is well established that biofilms of *P. aeruginosa* form in *CF* lungs *in vivo* (Moreau-Marquis et al., 2008). Perhaps growth in these biofilms protects the bacteria from exogenous oxidative stress, such as exposure to macrophages and antibiotics, and so also protects them against localized levels of oxidative stress. This is a difficult hypothesis to test as little is known about biologically relevant levels of oxidative stress in the lung. Reactive oxygen species like H_2_0_2_, for example, are highly reactive, making them a challenge to measure; estimates for *CF* lungs range from 0.07 to 5.00 μM and can vary according to the methods used (Worlitzsch et al., 1998; Jöbsis et al., 2000; Forman et al., 2016). Alternatively, while *CF* airways present a novel and stressful environment for *P. aeruginosa*, they may also be more temporally stable, without strongly fluctuating oxidative conditions. Either way, a downregulation of environmentally responsive oxidative stress systems in clinical isolates could occur, as they are no longer required to respond to fluctuations in oxygen availability in the new environment. This interpretation implies that the mutations generating a signal of positive selection in these genes result in loss of function associated with environmentally induced signals. Notably, the range of resistance to the exogenous oxidative stressors was wider for clinical than environmental isolates, suggesting either there may be different evolutionary pathways available to the strains for converging on this phenotype or the strains were isolated at different stages of adaptation to oxidative stress. These hypotheses are not mutually exclusive and deserve further, more focused attention.

### Antibiotic resistance of *CF* isolates is higher under anoxic conditions

Although *P. aeruginosa* has high levels of intrinsic antibiotic resistance (Pang et al., 2019), the persistent use of antibiotics to manage infections in *CF* patients often results in the evolution of even higher levels of resistance. On average, clinical and environmental isolates showed comparable levels of resistance to the commonly used antibiotics ciprofloxacin and tobramycin under oxic and microaerobic conditions, suggesting that environmental strains included in this study have very high levels of intrinsic antibiotic resistance. However, the range of resistance was wider for clinical strains, with some clinical isolates being substantially more resistant than environmental isolates under these conditions. The cause of this wide range of resistance among clinical isolates could be associated with the stage of infection: isolates from early infections would have resistance levels comparable to those from the environment whereas those from late-stage chronic infections would have much higher resistance levels. Unfortunately, this hypothesis cannot be tested with our data as we lack information on the stage of infection for these isolates. Nevertheless, under anoxic conditions, clinical strains are far more resistant on average than environmental strains (Figures 3A,B). This result suggests, in line with previous results (Field et al., 2005; Hill et al., 2005; King et al., 2010), that anoxic environments may be able to exaggerate, rather than weaken, selection for resistance to both antibiotics.

It has been suggested that reduced susceptibility to aminoglycosides under anoxic conditions stems from either *P. aeruginosa*’s reliance on oxidative phosphorylation to activate these transport systems (Hill et al., 2005; Schaible et al., 2012; Botelho et al., 2019), meaning these mechanisms would not work effectively in limited oxygen environments, or slow bacterial growth rates under anoxic conditions that impair the uptake of antibiotics (Hill et al., 2005; Borriello et al., 2006). Our results suggest neither of these interpretations is correct. We could find no evidence for reduced susceptibility to either antibiotic among environmental strains under anoxic conditions, as would be expected if oxidative phosphorylation-mediated transport systems were impaired. Moreover, cell densities for all strains – both clinical and environmental – were similar in anoxic environments (~10^7^ cells/mL), suggesting that the increased levels of resistance we observed among clinical isolates in anoxic conditions cannot be because they grow slower than environmental strains in this condition. Rather, our results point to strong selection for resistance under anoxic conditions in the *CF* lung. Whether this result is due to locally increased concentrations of antibiotics in anoxic regions of the *CF* lung or due to unrecognized mechanisms of interaction between anoxia and the mechanism of antibiotic action remains an important avenue for further investigation.

### Most additional patho-adaptive traits are insensitive to oxygen availability

Adaptation to the *CF* airways is often accompanied by loss of motility and virulence factors, along with the increased biofilm production (Smith et al., 2006; Folkesson et al., 2012; Sousa et al., 2018). We see similar results in our assays: clinical isolates had reduced motility and lower virulence factor production than environmental isolates, and these differences were largely unaffected by oxygen availability and phylogenetic relatedness. An interesting exception to this trend was in the increased ability of clinical isolates to form biofilms when oxygen levels are low, consistent with previous findings of biofilm formation increasing the antibiotic resistances of many bacterial species (Xu et al., 1998; Stewart and Franklin, 2008). Indeed, these results suggest that biofilm formation and antibiotic resistance in clinical isolates, unlike environmental isolates, is influenced by the availability of oxygen in the environment. Therefore, to fully understand the virulence of *P. aeruginosa* in *CF* infections, it appears to be important to gain a better understanding of biofilms and how they relate to antibiotic resistance under these differing oxygen conditions.

### Niche-specific phenotypes result from repeated evolution, not phylogeny

Evolution is a process of descent with modification, meaning that some strains or species could look alike not because they have evolved independently due to a shared selective environment but simply because they share a common ancestor. Evolutionary biologists have developed approaches to evaluate the contribution of shared ancestry to trait correlations that we have made use of here. These phylogenetically informed techniques are less commonly used in microbiology, perhaps because of the assumption that environmental sources of selection are usually so strong that they overwhelm any other potential sources of variation. Here, we have used one metric, phylogenetic heritability (*H*^2^), to quantify and account for trait values associated with shared inheritance in our analyses. A high phylogenetic heritability signal (i.e., *H*^2^ close to 1) indicates a strong signal of shared ancestry in trait variation across the phylogeny, suggesting these traits are either under strong stabilizing selection or subject to constraints that restrict the evolution of new phenotypes (Kamilar and Cooper, 2013). On the other hand, a lack of signal (i.e., *H*^2^ close to 0) indicates that traits evolve readily, leading to large differences among close relatives (Blomberg and Garland Jr., 2002; Losos, 2008; Kamilar and Cooper, 2013). Such a pattern is consistent with repeated evolution of the same trait independently of phylogeny, which is often taken to be a signal that those traits are under strong selection (Bailey et al., 2017).

Of the traits we investigated in this study, the vast majority show little evidence that phylogeny has a major influence on trait values. Moreover, in those traits where we did detect a statistically significant phylogenetic signal, the phylogenetic heritabilities were not high, such that accounting for phylogeny in our analyses usually had only marginal effects on trait values. Most importantly, phylogenetic corrections were small relative to the main effect of niche differences on trait values. In other words, niche differences between clinical and environmental isolates in these putative *CF* patho-adaptive traits are robust, even for traits with a moderate phylogenetic signal. This result lends support to the idea that *P. aeruginosa* can evolve rapidly and independently due to strong selection imposed by the conditions of the *CF* airway following colonization. That most new chronic infections of *CF* patients are derived from environmental sources, a result supported by the fact that chronic isolates in our study are spread across the phylogeny and not clustered in a single clade, lends further credence to the idea that the *CF* lung is an environment comprising novel selective pressures for most environmental strains (Schick and Kassen, 2018).

## Conclusion

It has long been known that phenotypic expression depends on the environment in which it is measured. This phenomenon is especially important when it comes to studies of parasites and pathogens, as measurements made on individuals grown outside of the host may have little bearing on their behavior and performance within the host. Historically, environmental context has been largely ignored in studies of human bacterial pathogens, and measurements made in lab environments have been used to infer disease prognosis. This situation certainly applies to understanding the phenotypic profile of *P. aeruginosa* isolates from *CF* patients, where there is only limited literature addressing trait changes thought to be associated with chronic infection under different oxygen conditions. We have addressed this gap here by providing insight into how patho-adaptive trait changes tied to chronic infection of *CF* airways differ among strains of *P. aeruginosa* grown under three different levels of oxygen availability. Our study suggests that some clinically important phenotypes such as antibiotic resistance and biofilm production are dependent on the oxygen condition in which they are measured. This observation may explain the lack of predictability offered by phenotypic characterization of clinical isolates for disease prognosis. Future work should examine the phenotypic traits of *P. aeruginosa* in microaerophilic and anoxic conditions, as well as their relationship to disease outcomes in these growth conditions, to gain a better understanding of chronic *CF* infections. The niche differences in clinical and environmental isolates for the putative *CF* patho-adaptive traits are robust, even for traits with a moderate phylogenetic signal, suggesting that *P. aeruginosa* in the *CF* lung are repeatedly experiencing novel selection pressures. Overall, our study indicates that oxygen availability and how it contributes to the adaptive evolution of *P. aeruginosa* within *CF* airways requires further investigation.

## Data availability statement

The original contributions presented in the study are included in the article/Supplementary material, further inquiries can be directed to the corresponding author.

## Author contributions

SS and RK designed the research and wrote the article. SS performed the research and analyzed the data. All authors contributed to the article and approved the submitted version.

## Funding

This work was supported by Cystic Fibrosis Canada (488978).

## Conflict of interest

The authors declare that the research was conducted in the absence of any commercial or financial relationships that could be construed as a potential conflict of interest.

## Publisher’s note

All claims expressed in this article are solely those of the authors and do not necessarily represent those of their affiliated organizations, or those of the publisher, the editors and the reviewers. Any product that may be evaluated in this article, or claim that may be made by its manufacturer, is not guaranteed or endorsed by the publisher.
